# Role of intraoperative patients positioning in endoscopic full-thickness resection of large gastric tumors under general anesthesia

**DOI:** 10.3389/fonc.2022.985257

**Published:** 2022-08-05

**Authors:** Li-Jun Zhou, Fei Xing, Dan Chen, Yan-Na Li, Shoaib Mohammad Rafiq

**Affiliations:** Department of Anesthesiology, Pain and Perioperative Medicine, the First Affiliated Hospital of Zhengzhou University, Zhengzhou, China

**Keywords:** Gastrointestinal Cancer, gastric tumors, submucosal tumors, endoscopic full-thickness resection, patient position, general anesthesia

## Abstract

Full thickness endoscopic resection of large submucosal gastric tumors (>3 cm) is a big challenge for endoscopists. Issues include how to efficiently resect the lesion, obtain homeostasis, and suture the defect. There are no guidelines regarding the importance of patient position on the success of endoscopic resections in anesthetized patients. Typically, the patient is placed in left lateral position for the endoscopic therapy and during the procedure patient’s position is changed to maintain the tumor above the gastric fluids to prevent gastric juices and tumor or tumor fragments from falling into the peritoneal cavity in the event of perforation. This study emphasized the importance of planning the procedure to ensure that the patient’s position and anesthetist’s concerns are met and allow optimal access to the lesion for endoscopic resection. Prior to sedation the patient should be positioned so that the tumor is in the up position which also prevents blood obscuring the operative field, helps detect bleeding points for immediately hemostasis. In addition, due to gravitational effect, the resected tumor will fall into the gastric cavity exposing the root of the tumor making resection easier and reduce procedure time. Preplanning avoids unnecessary readjustment of positioning and improves the ease and safety of the procedure.

## Introduction

Endoscopic full-thickness resection techniques are now widely applied in the management of large gastric submucosal tumors (>3 cm) (1–3). Although, endoscopic full-thickness resection is considered a safe and minimally invasive method, many challenging factors remain to be solved (4, 5). Endoscopist’s main focus is on the technical details of the resection including homeostasis and closure of the resulting defect (6, 7). To date, no study has focused on the critical effects of patient position on successful performance of these procedures and the left lateral positions and supine positions are considered the routine position for endoscopic procedures (8). In clinical practice, the tumor may be located anywhere in the stomach. When located on the dependent side it may be obscured by intragastric fluids which interfere with tumor resection, finding the tumor base, and achieving homeostasis.

Patient positioning for endoscopic procedures has not yet received much attention which contrasts with most other surgical procedures. However, the increasing requirement for general anesthesia for endoscopic resection procedures has led to the recognition of the need for patient positioning guidelines. The goal of optimal positioning is to provide the optimum access to the operative site while minimizing potential risks to the patient. Each position carries some degree of risk which is maximized in the anaesthetized patient who cannot make others aware of compromised conditions. Patients may also be transferred and positioned on operating tables whilst they are unconscious. The maneuvering and the final positioning have an impact on potential injuries sustained under anesthesia as endotracheal tubes, intravascular lines, and urinary catheters should be free to move and adequately secured before any movement. This all adds to challenges encountered by both the endoscopist and anesthetist attempting changing patient position during the procedure.

Because of the importance of keeping the tumor at a high position away from the dependent portion of the operative field, it is clear that the patient’s position should vary according to the location of tumor. The optimal patient position can be left lateral, right lateral, prone, or even supine depending on the location of tumor. If unclear, the location of the tumor can be confirmed by CT before the procedure. This study discusses the influence of patient position on the success of large submucosal gastric tumor resections.

## Importance of patient positioning for endoscopic resection

The left lateral position remains the standard (traditional) position for endoscopic therapy. In our early practice, we used the left lateral position as the routine position. However, in some cases, we noted that homeostasis was difficult because blood immediately covered the operating field obscuring the bleeding point resulting in a delay in achieving hemostasis. We also found that it was sometimes difficult to find the base of tumor when fluid covered the operating field.

After group discussion, it was recognized that it was possible to use the effects of gravity to overcome these difficulties. Ensuring that the tumor was located above the dependent part of the gastric cavity resulted in fluids and the tumor tending to move to the dependent portion away from the operative field. In addition, as resection proceded the tumor fell into the gastric lumen allowing the base to be easily seen and bleeding points addressed. The solution was to initially position of the patient depending on the location of the tumor.

**Figure 1:**
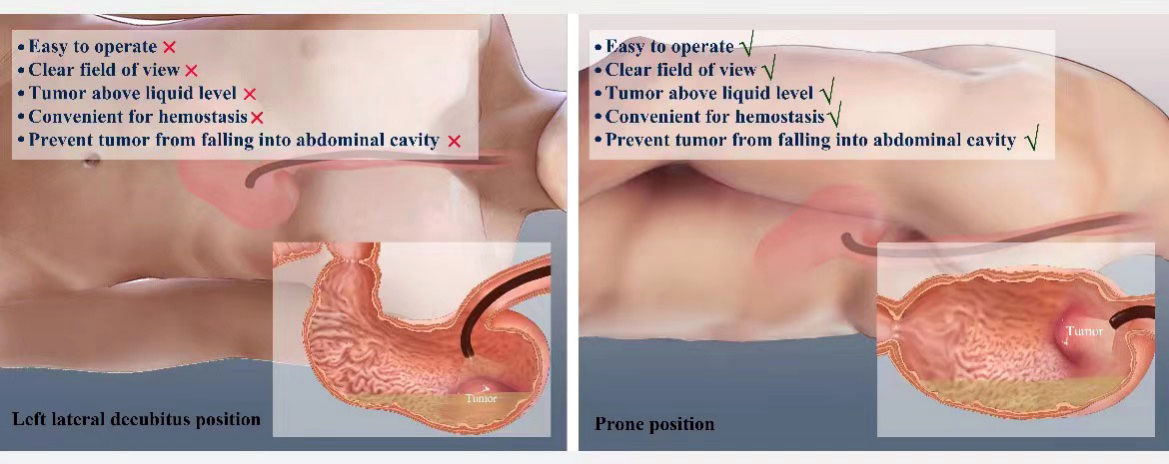
Graphical presentation of patient position variation during the Endoscopic full-thickness resection.

Liu et al. described a typical case of a 56-year-old women whose CT scan suggested a tumor on the posterior wall of greater curvature of the stomach (9). The endoscope was inserted into the stomach with the patient in the left lateral position. After washing the stomach, it was noted that watery fluids covered the tumor. The patient’s position was changed to the prone position which produced an unobscured endoscopic field. The tumor was resected and the resected tumor fell into the gastric cavity away from the operative field allowing the defect to be closed using endoloops and endoclips without leakage of fluids into the peritoneal cavity (**Figure 1**).

According to our experience the combination of endoscopy and CT scan can usually provide the required information about the location of the tumor and the effects of patient position (10–12) allowing the patient position to be pre-planned. The supine position (include anterior lateral) and left lateral position is convenient for most patients requiring endoscopic full-thickness resections. For patients where the tumor is located on the posterior wall and greater curvature of gastric body and fundus, the prone position or right lateral is best. When in doubt and before the endoscopic resection is begun, water can be injected into the gastric cavity to confirm that the position chosen is ideal. If not the position can be changed allowing safe endoscopic full-thickness resection.

## Importance of patient positioning from anesthetist’s perspective

For surgical procedures, patients are placed on an operating table, and for the endoscopic treatment, on a trolley. There are well-established guidelines for patient positioning during surgical procedures, but no such guidelines are available for endoscopic therapies, especially when resecting large gastric tumors (13, 14). Advanced endoscopic techniques (eg, endoscopic submucosal dissection, endoscopic full-thickness resections, and peroral endoscopic myotomy) may have procedure times exceeding 60 minutes and in some cases lasting several hours, and thus frequently require general anesthesia (15–17). When general anesthesia is intended, the airway is typically secured while the patient is still supine *via* an endotracheal tube. If the patient position needs to be adjusted during endoscopy therapy, extra care must be taken to secure and tape the endotracheal tube to prevent dislodgement while the patient is left lateral or prone or during position changes. Placing an anesthetized patient in the prone position requires the coordination of the entire staff (endoscopist, anesthetist, nurses). The primary duty of anesthesiologist is to coordinate the maneuver while maintaining inline stabilization of the cervical spine and monitoring the endotracheal tube. In order to prevent dislodging, the endotracheal tube should be disconnected from the circuit before shifting from supine to prone. Which, and how many, lines and monitors are disconnected during the shifting is up to the clinical judgment of the anesthesiologist. Ventilation and monitoring should be resumed as rapidly as possible. Patient positioning is critically important and should be preplanned by the endoscopist to avoid during the procedure changes in position and possible complication associated with it.

Frequently, the patient can assist in positioning prior to induction of anesthesia. However, the operating room team must move the patient while the patient is under general anesthesia. Additional staff will be needed for the transfer and positioning of patients with morbid obesity or unstable spine fractures. The anesthesiologist must be aware of any blood pressure alterations when the patient is moved following the induction of general anesthesia and must ensure a safe systemic blood pressure prior to any patient movement.

## Future perspective and conclusion

Endoscopic full-thickness resection of large submucosal tumors is a recent development (18, 19). Most studies have focused on technical aspects such as how to resect the lesions, deal with homeostasis, and suturing of the defect (20–24). To our knowledge, no study has been primarily concerned with patient positioning for endoscopic full-thickness resections.

Patient position is a critical element for the successful endoscopic resection therapy of large submucosal gastric tumors. Prior to achieving any position, the patient must be transferred onto the endoscopy room trolley. The final position of the patient is of the utmost importance, but achieving these positions requires careful planning and collaboration. The overall plan for each patient position should be discussed prior to any procedure.

The ideal position depends on the location of the tumor and should be one in which the tumor is not covered by fluid and can fall into the gastric lumen away from the operative field. The combination of CT and endoscopic examination can help to make a primary plan for the patient’s position. Even during procedure, one can inject water and observe the operative field and make sure that tumor is at higher level position.

Patient positioning should be individualized according the location of the tumor. Traditionally, the left lateral position and supine position are used for the tumor resections. After the stomach cleansing, the potential operative field (tumor) area to ascertain whether it is partially or fully covered with gastric fluids. If so, the patient’s position is changed from left lateral to right lateral, supine or prone position depending on the location of the tumor so as to keep the tumor away from the dependent portion of the stomach. Typically, if the tumor is located in the lesser curvature of stomach, the left lateral position is used. For tumors located along the greater curvature. The right lateral position can be used with the supine position being used for tumors located in the anterior gastric wall and the prone position for tumors located in the posterior gastric wall.

## Data availability statement

Publicly available datasets were analyzed in this study. This data can be found here: This is a perspective article. No original data was produced for this article. However, current data collected for writing this article is available with the corresponding author. It can be provided on request. (E-mail: 529358809@qq.com).

## Ethics statement

This study was reviewed and approved by the independent ethics committee of the First Affiliated Hospital of Zhengzhou University. Written informed consent was obtained from all participants for their participation in this study.

## Author contributions

Study concept and design: L-JZ, FX. Manuscript writing: L-JZ, FX. Analysis and interpretation of data: DC, Y-NL, SR. Administrative, technical or material support: DC, Y-NL, SR. Critical revision of manuscript: L-JZ, SR. All authors contributed to the article and approved the submitted version.

## Conflict of interest

The authors declare that the research was conducted in the absence of any commercial or financial relationships that could be construed as a potential conflict of interest.

## Publisher’s note

All claims expressed in this article are solely those of the authors and do not necessarily represent those of their affiliated organizations, or those of the publisher, the editors and the reviewers. Any product that may be evaluated in this article, or claim that may be made by its manufacturer, is not guaranteed or endorsed by the publisher.
